# Data evaluation of broiler chicken rearing and slaughter—An exploratory study

**DOI:** 10.3389/fvets.2022.957786

**Published:** 2022-10-06

**Authors:** Annika Junghans, Lea Deseniß, Helen Louton

**Affiliations:** Animal Health and Animal Welfare, Faculty of Agricultural and Environmental Sciences, University of Rostock, Rostock, Germany

**Keywords:** poultry, mortality, condemnation, slaughter, rejection

## Abstract

To process and evaluate the data from broiler fattening and slaughtering, we investigated the production data of 107 straight run flocks of the commercial meat-type breed Ross 308 (Aviagen, EU). All flocks were raised and slaughtered in Germany and the average slaughter age was 37 days. The health outcomes of interest were mortality, average weight, and the slaughter results. First-week mortality, cumulative mortality, stocking density, flock size, season, production week of the parental flock, farm, antibiotic treatment, and the interaction between antibiotic treatment and season were considered as possible influencing factors. The average first-week mortality (FWM) and cumulative mortality percentages were 0.66 and 2.74%, respectively. First-week mortality was influenced by flock size, production week of the parental flock, and the interaction between antibiotic treatment and season, whereas cumulative mortality was influenced by antibiotic treatment, farm, and first-week mortality. The average weight (mean 2.30 kg) was influenced by season, stocking density, flock size, farm, and the interaction between antibiotic treatment and season. The condemnation rate was on average 1.48%, with the most common causes being deep dermatitis (mean 0.63%), ascites (mean 0.53%), and not suitable for production/general disease (mean 0.25%). Several factors influenced the causes of condemnation, with season being the most predominant one, followed by the interaction between antibiotic treatment and season, the antibiotic treatment alone, and stocking density.

## Introduction

Although vegetarian nutrition is becoming more popular these days, broiler meat is still favored because it is low in fat, easy to prepare, and affordable. The German meat industry produced 623,165,170 broilers in 2020, corresponding to a weight of 1,066,528,075 tons (1). Because broiler meat is an essential part of the meat produced in Europe and worldwide, the enormous effort put into research on broiler welfare is explainable and reasonable (2).

For poultry production, mortality records are of major importance because they may reflect possible disease incidences (3). Furthermore, performance and health data of broiler flocks must be collected regularly during the fattening period (4). In Germany, mortality must be routinely recorded in broiler flocks according to Article 19 of the German Order on the Protection of Animals and the Keeping of Production Animals (5). This regulation requires the farmer to document the daily mortality rate and calculate the cumulative mortality rate (5). The first-week mortality (FWM) in the life of broilers is a significant factor, which is widely assessed in poultry production (6) and can serve as an indicator of the health status and performance of the broiler flock during the fattening period (7). Therefore, there is a need to put more effort into the first 7 days of the chicks' life to make sure they develop good drinking and feeding behavior and thus quickly maximize their opportunity for growth. However, there are changes in the weekly mortality percentages throughout the fattening process (7). Because large numbers of broilers are processed, even incidents of small effects during the fattening period can be of large economic importance to the meat industry (8). Thus, high mortality in broiler flocks is associated with a lower income for the broiler farmers (7) and a considerable financial loss caused by mortality and injuries from the enormous number of broilers that are slaughtered, which represents a lack in animal welfare that must be addressed (9).

At the time of slaughter, the weight of the animals is recorded and can therefore be regarded as a standardized and objective measure of the health of a broiler flock; furthermore, the weight data can reveal poor flock uniformity (10). At the processing plant, the carcasses and associated by-products undergo a final post-mortem inspection. All external surfaces, body cavities, and by-products are examined and the findings recorded (11). Those recordings can be used for surveillance purposes. The feedback the farmer receives from slaughterhouses for each flock includes information on flock performance, condemnation rate, and causes of condemnation. By use of these data, improvements in the production chain can be made, not only by the farmer to enhance the production but also by the veterinarians for advising and consulting or by the personnel and veterinarians working at the processing plant, who perform a risk-based meat inspection. In addition, this information allows scientists to improve their fields of study (12). Several authors have already researched possible factors that may influence the results of broiler fattening. Van Limbergen et al. (13) showed in their study the influence of many factors of broiler farm management and housing on broiler health and performance, as well as the impact of health problems caused by septicemia, coccidiosis, and dysbacteriosis. De Jong and van Riel (4) found that causes of condemnation at slaughter, uniformity of carcass weight, first-week mortality, and cumulative mortality all showed seasonal variations, with the best performances obtained when the broilers were farmed during the summer months.

Mean values of 1.36% (12) and 1.10% (14) were reported as condemnation percentages in broilers, and Salines et al. (12) mentioned generalized constipation, cachexia, and non-purulent skin lesions as the main findings in meat inspection. In addition, Alfifi et al. (14) found scratching and dermatitis as the most common reasons for condemnation, followed by ascites.

The objective of this study was to combine data on broiler chicken rearing and slaughtering from the same flocks and to identify factors that may influence the mortality during the fattening period, the slaughter weight, and the causes of condemnation recorded at the processing plant. This enabled the identification of farm management factors to inform poultry production best practices. This study provides an overview of current broiler rearing and slaughter data in an average German broiler production system.

## Materials and methods

### Study population and design

For this study, flocks from two farms in Germany were analyzed: five barns from farm 1 and six barns from farm 2 were included in the study. Some flocks had to be excluded from the study because of missing data. Both farms belonged to the same company, were in the same area, only 18 km apart from each other, and were led by the same operation manager.

#### Barns

All barns had a small room serving as a sluice and were entered with farm-specific clothes and barn-specific shoes. All barns were emptied, cleaned, and disinfected in the service period between the fattening periods. The authorized broiler numbers for the barns were 10,100 (barn 1, farm 1), 9,700 (barn 2, farm 1), 10,100 (barn 3 and 4, farm 1), 29,200 (barn 5, farm 1), 41,500 (barn 1–4 and 6 of farm 2), 19,300 (barn 5, farm 2) with an average 27,000 birds per flock. All barns were equipped with round feeders and feed was supplied *ad libitum*. The feeder space differed according to the number of birds housed, resulting in feeder space per bird according to legal regulation. There was a difference in the litter material used on the two farms; farm 1 used straw granulate for bedding, and farm 2 used straw pellets.

To keep the temperature in the desired range, all barns were equipped with spray cooling systems and a gas heating system. Before housing, the barns were heated up to an air temperature of 33°C. All barns were closed barns with a forced ventilation system. The ventilation capacity ranged between 6.7 to 14.5 m^3^ per housed animal and hour and was adapted to the size of the birds and their necessity by a temperature and humidity control system.

Both farms received their chicks from the same hatchery and the proximity from hatchery to farm was similar (287 km for farm 1 and 276 km for farm 2). Both farms used the same vaccination program: vaccination program against Infectious Bronchitis Virus (IBV) where a primer (half dose) and booster (full doses) were applied at the hatchery and at day 10 on the farm, Newcastle Disease Virus (NDV) and Infectious Bursal Disease (IBD, also known as Gumboro) were applied *via* drinking water at day 16 on the farm.

#### Data generation

The first chicks were housed in January 2019 and the last in June 2020, and 107 fattening periods were considered in the study. The following number of fattening periods were included for each barn: barn 1, farm 1: 8; barn 2 farm 1: 8, barn 3, farm 1: 8; barn 4 farm 1: 8, barn 5, farm 1: 9; barn 1 farm 2: 11, barn 2, farm 2: 11; barn 3 farm 2: 11, barn 4 farm 2: 11; barn 5 farm 2: 11; barn 6 farm 2: 11. The mean stocking density was 33.86 kg/m^2^, with a minimum of 30.08 and a maximum of 39.43 kg/m^2^. For further analysis, the flock size was categorized into small ( ≤ 11,000 broilers), medium (>11,000 to ≤ 30,000 broilers), and large (>30,000 to ≤ 42,000 broilers). Regarding the genotype, only Ross 308 (Aviagen, EU) broilers were included in this study. All flocks were of straight run. Only birds from the final depopulation were included in the study, and the average duration of the fattening period was 37 days (minimum 35 days and maximum 40 days).

The catching time was evaluated and was divided into day (>4 am until < 9 pm) and night (≥9 pm until ≤ 4 am). In total, 82 flocks were caught at night time and 25 flocks were caught during the day. All birds were brought to the same processing plant with a transport distance from farm 1 of 229 km and 242 km from farm 2. Slaughtering took place from February 2019 to July 2020, using a controlled atmosphere stunning as the stunning method. The meat inspection was done by official authorities and the staff of the processing plant, according to Commission Implementing Regulation (EU) 2019/627 (11).

To analyze the influence of the seasons, the following seasons were used: winter (= December, January, February), spring (= March, April, May), summer (= June, July, August), and fall (= September, October, November). The production week of the parental flock was also included in the study and varied from 3 to 38 weeks.

The information and data used in this study were obtained from the following four sources: (i) farm record: date of housing, number of chicks per flock (flock size), mortality (FWM and cumulative mortality), litter material, catching time; (ii) delivery note: production week of the parental flock, genotype; (iii) delivery and application documents from the veterinarian: antibiotic treatment (name of the drug, date of application, number of days of usage, age of the broilers when the drug was given, diagnosis); (iv) slaughter records: date of slaughter, average weight, number of birds processed, condemnation rate, dead-on-arrival (DOA) rate, and causes of condemnation. The latter included deep dermatitis, ascites, not suitable for production/general disease, hepatic changes, polyserositis, underdevelopment/emaciation, other pathologic findings/hematoma/injuries, and changes in color/odor/texture. Before the start of each antibiotic treatment, a clinical examination of the flock was performed by a veterinarian. The clinical diagnoses were used, together with pathological findings of dead or culled birds, a microbiological examination and antibiogram were carried out to find the most suitable antibiotic. All microbiological examinations and antibiograms were performed at the laboratory of the veterinarian in charge and the data sets were provided by the farmer.

### Statistical analysis

All information about the broilers, considering the whole fattening period and the details about the slaughtering of the flocks, was provided by the farmer and handed in on paper. The data were collected and transformed into an Excel table and then transferred to IBM SPSS Statistics software, version 27 (SPSS Inc., Chicago, IL, United States). Data inputs were validated and descriptive statistics were obtained to validate the information, followed by a calculation of the means, the minimum and maximum values, and the standard deviation. Histograms on all relevant farm-level and slaughter variables were conducted and checked visually for potential errors, extreme values, and normal distribution.

**Figure 1:**
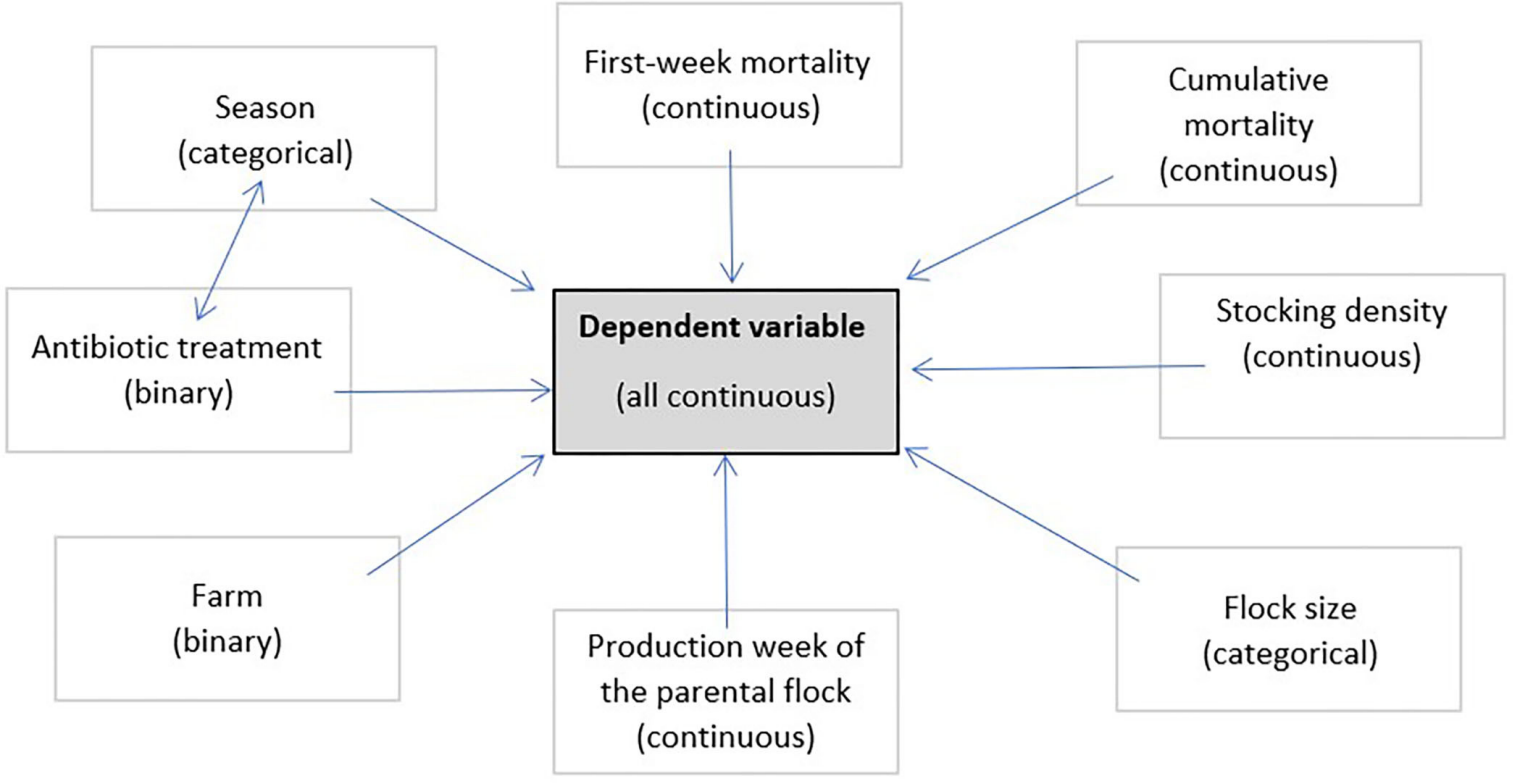
Overview of the independent variables and their characteristics used in the multivariable model. First-week mortality, cumulative mortality, average weight, and the causes of condemnation (condemnation rate in total, DOA rate, deep dermatitis, ascites, not suitable for production/general disease, hepatic changes, polyserositis, underdevelopment/emaciation, other pathologic findings/hematoma/injuries, and changes in color/odor/texture) were each used as the dependent variable.

All data provided were checked for their influence on the dependent variables and independent variables with at least one significant *p*-value ( ≤ 0.05) were included in the model. This led to eight independent variables plus the interaction between season and antibiotic treatment as a random effect (Figure 1). The farm was included in the model to consider any influence of the difference in the litter that was used. First-week mortality, cumulative mortality, average weight, and the causes of condemnation (condemnation rate in total, DOA rate, deep dermatitis, ascites, not suitable for production/general disease, hepatic changes, polyserositis, underdevelopment/emaciation, other pathologic findings/hematoma/injuries, and changes in color/odor/texture) were each used in a multivariable model as the dependent variable. For a more precise model, backward selection was performed with regard to the corrected *R*^2^. The model with the highest *R*^2^ was chosen for further interpretation. To check the influence of the catching period, an ANOVA was additionally performed and evaluated for the condemnation rate in total and the DOA rate. For the ascites findings, an additional ANOVA was performed to check the influence of the average weight.

*P* ≤ 0.05 were considered significant. By using the conditional studentized residual plots, the residuals and the assumptions of homogeneity of variance were predicted and checked visually for normal distribution.

## Results

### Mortality

The descriptive results for FWM, cumulative mortality, and average weight are presented in Table 1. The overall mean first-week mortality was 0.66%. As shown in Table 2, the FWM was influenced by flock size (*p* < 0.001), production week of the parental flock (*p* = 0.044), and the interaction between antibiotic treatment and season (*p* = 0.004). Smaller flock sizes and parental flocks in an earlier production week led to a lower FWM. In addition, the FWM was higher in flocks without reported antibiotic treatment (0.67%) than in flocks reported to have been treated with antibiotics (0.64%).

**Table 1:** Mean values and standard deviation of first-week mortality, cumulative mortality, and average weight, separately listed according to season and use of antibiotic treatment.

	***n***	**First-week mortality (%)**	**Cumulative mortality (%)**	**Average weight (kg)**
**Season**				
Fall	16	0.46 ± 0.11	2.20 ± 0.67	2.38 ± 0.09
Winter	23	0.69 ± 0.27	2.69 ± 0.65	2.31 ± 0.12
Spring	41	0.72 ± 0.30	3.15 ± 1.79	2.27 ± 0.10
Summer	27	0.66 ± 0.24	2.47 ± 1.21	2.30 ± 0.17
**Antibiotic treatment**				
No	77	0.67 ± 0.25	2.52 ± 1.32	2.33 ± 0.12
Yes	30	0.64 ± 0.30	3.28 ± 1.31	2.24 ± 0.12
Total	107	0.66 ± 0.28	2.74 ± 1.36	2.30 ± 0.13

n, number of examined flocks.

**Table 2:** *P*-values of the multivariate analysis using a generalized linear model for the dependent variables first-week mortality, cumulative mortality, and average weight.

**Independent variable**	**Dependent variable**		
	**First-week mortality**	**Cumulative mortality**	**Average weight**
	***R*^2^** **0.284**	***R*^2^** **0.185**	***R*^2^** **0.648**
Season	–	–	0.002
First-week mortality		0.003	–
Cumulative mortality	–		0.085
Stocking density	–	–	< 0.001
Flock size	< 0.001	0.054	0.002
Production week of the parental flock	0.044	–	–
Antibiotic treatment	–	0.026	–
Farm	–	0.008	0.003
Interaction Antibiotic treatment * Season	0.004	–	< 0.001

The overall mean cumulative mortality was 2.74%. Considering the seasons, the highest rate was observed in spring (3.15%) and the lowest in fall (2.20%), but seasonal variations observed did not differ and were thus excluded from the final model. Flocks that had been treated with antibiotics had higher cumulative mortality (3.28%) than flocks that had not been treated (2.52%) (*p* = 0.026; Table 2). The statistical model showed an influence of FWM (*p* = 0.003), farm (*p* = 0.008), and antibiotic treatment (*p* = 0.026) on cumulative mortality (Table 2).

### Average weight

The average weight of the broilers of all flocks was 2.30 kg (Table 1). Considering the seasons, the heaviest birds were found in fall (mean 2.38 kg) and the lightest in spring (mean 2.27 kg) (*p* = 0.002; Table 2). The average weight was higher in the flocks that had not been treated with antibiotics (mean 2.33 kg) than in those that had been treated (mean 2.24 kg), the difference was, however, not significant, only the interaction between antibiotic treatment and season was supported (*p* < 0.001). Stocking density (*p* < 0.001), farm (*p* = 0.003), and flock size (*p* = 0.002) also had an effect on the average weight (Table 2). A higher stocking density was associated with a higher average weight. Flocks categorized as large had a lower average weight than flocks categorized as small, while medium flocks had the highest average weight.

### Causes of condemnation

The condemnation percentage in our study was 1.48%, with deep dermatitis (mean 0.63%) and ascites (mean 0.53%) being the major causes of condemnation (Table 3). The condemnation percentage was influenced by the season (*p* < 0.001), the production week of the parental flock (*p* = 0.008), and the use of antibiotics (*p* = 0.002; Table 4). The highest condemnation percentages were found in fall (mean 1.90%) and the lowest in winter (mean 1.34%) and spring (1.31%). The DOA percentage in our study showed a mean value of 0.17% and was influenced by FWM (*p* = 0.039), flock size (*p* = 0.046), the use of antibiotics (*p* = 0.018), and the interaction between antibiotic treatment and season (*p* = 0.040; Table 4). Flocks with a high FWM had a higher DOA percentage. Flocks without antibiotic treatment had lower DOA percentages than flocks with antibiotic treatment. An influence of the catching time was found in the additional ANOVA, showing that flocks that were caught during the night time had a lower DOA percentage compared to the flocks caught during day time (*p* < 0.001). Deep dermatitis was the most common cause of condemnation during slaughter in our study and was influenced by the season (*p* = 0.021) and the production week of the parental flock (*p* = 0.002; Table 4). The pathological finding was that ascites examined during slaughter was strongly influenced by the season (*p* < 0.001), the flock size (*p* = 0.002), antibiotic treatment (*p* < 0.001), and the interaction between antibiotic treatment and season (*p* < 0.001; Table 4). Differences between the seasons on the ascites findings could be seen, with the highest findings in winter (mean 0.71%) and fall (mean 0.61%). In spring (mean 0.45%) and summer (0.43%), fewer broiler carcasses were condemned because of ascites. The results of the ANOVA with the influence of average weight on ascites showed an influence of average weight (*p* = 0.038). The pathological findings were that not suitable for production/general disease (*p* < 0.001) and hepatic changes (*p* = 0.001) were both influenced by the stocking density, and another finding was that not suitable for production/general disease was additionally influenced by the season (*p* = 0.044). Although polyserositis was only recorded in 0.09% of the condemned carcasses (Table 3), the multivariate model revealed influences for the factors season (*p* < 0.001), antibiotic treatment (*p* < 0.001), and the interaction between antibiotic treatment and season (*p* < 0.001; Table 5). Underdevelopment/emaciation was influenced by season (*p* = 0.010) and by stocking density (*p* < 0.001), whereas other pathologic findings/hematoma/injuries were influenced by season (*p* = 0.014), cumulative mortality (*p* = 0.015), production week of the parental flock (*p* < 0.001), and the interaction between antibiotic treatment and season (*p* = 0.021; Table 5). The least common finding (changes in color/odor/texture: mean value of 0.04%; Table 3) was influenced by season (*p* = 0.012), stocking density (*p* = 0.048), flock size (*p* = 0.013), antibiotic treatment (*p* = 0.022), and the interaction between antibiotic treatment and season (*p* = 0.018; Table 5).

**Table 3:** Causes of condemnation (%), mean, minimum, maximum, and standard deviation (SD).

**Cause of condemnation**	***n***	**Mean**	**Minimum**	**Maximum**	**SD**
Condemnation rate in total	107	1.48	0.12	4.63	0.89
Deep dermatitis	106	0.63	0.04	3.13	0.67
Ascites	106	0.53	0.19	0.53	0.28
Not suitable for production/general disease	107	0.25	0.00	1.66	0.23
Dead on arrival	107	0.17	0.03	0.66	0.09
Hepatic changes	106	0.11	0.00	0.62	0.09
Polyserositis	106	0.09	0.00	2.10	0.25
Underdevelopment/emaciation	106	0.05	0.00	0.41	0.07
Other pathologic findings/hematoma/injuries	106	0.05	0.00	0.30	0.04
Changes in color/odor/texture	106	0.04	0.00	0.30	0.04

n, number of examined flocks.

**Table 4:** *P*-values of the multifactorial analysis using a generalized linear model for the dependent variables condemnation rate in total, dead-on-arrival (DOA), deep dermatitis, ascites, not suitable for production/general disease, and hepatic changes.

**Independent variable**	**Dependent variable**					
	**Condemnation rate in total**	**DOA**	**Deep dermatitis**	**Ascites**	**Not suitable for production/general disease**	**Hepatic changes**
	***R*^2^** **0.174**	***R*^2^** **0.197**	***R*^2^** **0.146**	***R*^2^** **0.392**	***R*^2^** **0.206**	***R*^2^** **0.207**
Season	< 0.001	0.167	0.021	< 0.001	0.044	0.062
First-week mortality	0.133	0.039	–	–	0.337	–
Cumulative mortality	–	0.088	–	–	0.128	0.187
Stocking density	0.162	0.139	0.133	0.226	< 0.001	< 0.001
Flock size	–	0.046	–	0.002	0.068	0.102
Production week of the parental flock	0.008	0.236	0.002	–	–	–
Antibiotic treatment	0.002	0.018	0.285	< 0.001	0.207	0.106
Farm	–	0.198	–	–	–	–
Interaction antibiotic treatment * season	–	0.040	–	< 0.001	–	–

**Table 5:** *P*-values of the multifactorial analysis using a generalized linear model for the dependent variables polyserositis, underdevelopment/emaciation, other pathologic findings/hematoma/injuries and changes in color/odor/texture.

**Independent variable**	**Dependent variable**			
	**Polyserositis**	**Underdevelopment/emaciation**	**Other pathologic findings/hematoma/injuries**	**Changes in color/odor/texture**
	***R*^2^** **0.278**	***R*^2^** **0.192**	***R*^2^** **0.331**	***R*^2^** **0.217**
Season	< 0.001	0.010	0.014	0.012
First-week mortality	–	–	–	–
Cumulative mortality	0.147	0.224	0.015	–
Stocking density	–	< 0.001	0.109	0.048
Flock size	–	0.227	0.146	0.013
Production week of the parental flock	–	0.168	< 0.001	–
Antibiotic treatment	< 0.001	–		0.022
Farm	0.272-	–	–	–
Interaction antibiotic treatment * season	< 0.001	–	0.021	0.018

### Antibiotic treatment

Regarding the antibiotic treatment, 30 of the 107 flocks were reported to have been treated with antibiotics during the fattening period, whereas 77 had not been treated (Table 6). Of the 30 flocks with antibiotic treatment, 25 were categorized as large flocks (>30,000 to ≤ 42,000 broilers), and 5 flocks were categorized as medium ones (>11,000 to ≤ 30,000 broilers). None of the small flocks ( ≤ 11,000 broilers) had been treated. We observed differences in the seasons, with most flocks with antibiotic treatment housed in spring (*n* = 18), whereas fewer flocks received antibiotic treatment in winter (*n* = 8), summer (*n* = 3), and fall (*n* = 1). Eight flocks were treated two times within the fattening period (one flock in fall, one flock in summer, and six flocks in winter), and two flocks were treated three times (both in spring). The other 20 flocks were treated one time with antibiotics during the fattening period.

**Table 6:** Antibiotic treatments (*n* = 30): name, active ingredient, diagnosis, withdrawal time of drug (days), number of treatments, week of life when treated, season, and size of the treated flock.

**Antibiotic name**	**Active ingredient**	**Diagnosis**	**Withdrawal time of drug (d)**	**Number of treat-ments**	**Week of life when treated**	**Season**	**Flock size**
Ampiciph	Ampicillin	A	6	1	3	Winter	Large
Metaxol	Trimethoprim, sulfamethoxazole	UYSI	5	1	3	Winter	Medium
Metaxol	Trimethoprim, sulfamethoxazole	UYSI	5	1	1	Spring	Large
Metaxol	Trimethoprim, sulfamethoxazole	UYSI	5	1	1	Spring	Large
Metaxol	Trimethoprim, sulfamethoxazole	UYSI	5	1	1	Spring	Large
Metaxol	Trimethoprim, sulfamethoxazole	UYSI	5	1	1	Spring	Medium
Metaxol	Trimethoprim, sulfamethoxazole	UYSI	5		1	Spring	Large
Belacol	Colistin sulfate	*E. coli* infection	2		4		
Octacillin	Amoxicillin	E	1		5		
Metaxol	Trimethoprim, sulfamethoxazole	UYSI	5	1	1	Spring	Large
Phenocillin	Phenoxymethyl-penicillin	E	2	1	2	Spring	Large
Metaxol	Trimethoprim, sulfamethoxazole	UYSI	5	1	2	Summer	Large
Metaxol	Trimethoprim, sulfamethoxazole	UYSI	5	1	3	Summer	Large
Fluonix	Enrofloxacin	Polyserositis	7	2	3	Summer	Large
Metaxol	Trimethoprim, sulfamethoxazole	UYSI	5		2		
Belacol	Colistin sulfate	*E. coli* infection	2	2	5	Fall	Medium
Suramox	Amoxicillin	A	1		5		
Octacillin	Amoxicillin	*E. coli* infection	1	2	5	Winter	Large
Belacol	Colistin sulfate	A	2		4		
Octacillin	Amoxicillin	*E. coli* infection	1	2	5	Winter	Large
Belacol	Colistin sulfate	A	2		4		
Octacillin	Amoxicillin	*E. coli* infection	1	2	5	Winter	Large
Belacol	Colistin sulfate	A	2		4		
Ampiciph	Ampicillin-	A	6	2	3	Winter	Large
Belacol	Colistin sulfate	*E. coli* infection	2		5		
Ampiciph	Ampicillin-	A	6	2	3	Winter	Large
Belacol	Colistin sulfate	*E. coli* infection	2		4		
Ampiciph	Ampicillin-	A	6	2	3	Winter	Large
Belacol	Colistin sulfate	*E. coli* infection	2		5		
Lincospectin	Lincomycin, spectinomycin	E	5	1	1	Spring	Large
Lincospectin	Lincomycin, spectinomycin	E	5	1	1	Spring	Large
Octacillin	Amoxicillin	A	1	1	5	Spring	Large
Octacillin	Amoxicillin	A	1	1	3	Spring	Medium
Belacol	Colistin sulfate	*E. coli* infection	2	3	3	Spring	Large
Octacillin	Amoxicillin	A	1		4		
Octacillin	Amoxicillin	A	1		5		
Lincospectin	Lincomycin, spectinomycin	E	5	1	1	Spring	Large
Lincospectin	Lincomycin, spectinomycin	E	5	1	1	Spring	Large
Lincospectin	Lincomycin, spectinomycin	E	5	1	1	Spring	Large
Lincospectin	Lincomycin, spectinomycin	E	5	1	1	Spring	Large
Lincospectin	Lincomycin, spectinomycin	E	5	1	1	Spring	Large
Lincospectin	Lincomycin, spectinomycin	E	5	1	1	Spring	Medium

E, Enteritis; A, Arthritis; UYSI, Umbilical yolk sac inflammation; *E. coli*, Escherichia coli. The flock size was categorized as follows. Small: ≤ 11,000 broilers; medium: >11,000 to ≤ 30,000 broilers; large: >30,000 to ≤ 42,000 broilers.

## Discussion

This study evaluated flock production and slaughter records and assessed factors influencing various production metrics including FWM, cumulative mortality, average weight, and condemnation percentages. Our analysis is suggestive that FWM (mean: 0.66%, *n* = 107 flock cycles) was influenced by flock size, production week of parental flock, and the interaction between antibiotic treatment and season. The interaction between antibiotic treatment and season described whether antibiotics were used in the flock during the fattening period and the differences between the number of treatments per season and its effect on the FWM. Previous studies reported higher percentages of 0.94, 1.03, 1.10, and 1.82% than we observed (6, 13, 15, 16). The relatively low FWM in the flocks of our study might suggest that the chicks were of good quality and health, and the management and brooding conditions were good. According to Yerpes et al. (6), the FWM can be used as an important production criterion in poultry production. In our study, the first-week mortality differed between flocks, and several influencing factors were identified, underlining the statement by Yerpes et al. (6). Regarding the factors which can influence first-week mortality in broilers, van Limbergen et al. (13) cited floor quality, ventilation type, presence of other professional activities of the farmer and neonatal septicemia as the most common ones. In addition, Heier et al. (17) described a relationship between stocking density and FWM, whereby flocks with higher stocking density had lower mortality in the first week after housing. In our study, the stocking density did not affect first-week mortality, but the flock size did. The highest FWM was observed in flocks categorized as medium while the lowest was found in flocks categorized as small. This could be because smaller flocks had a lower infection pressure and the broilers may also have less stress due to the smaller number of birds. Yerpes et al. (6) identified the age of the parental flock, gender of the chicks, genotype, type of broiler housing, presence of drip cup, egg storage, and study year as factors influencing the FWM. An influence of the production week of the parental flock was also observed in our study: the FWM increased if the production week of the parental flock increased, also confirming the findings of O'Dea et al. (18). These authors described a higher mortality in broiler chicks produced by 57-week-old parental flocks than in those produced by 28- and 43-week-old parental flocks. After the broilers reached the age of 3 weeks until the time of slaughter, the cumulative mortality in all flocks was the same, regardless of the age of the parental flocks (18). These results were similar to ours. In contrast, McNaugton et al. (19) found that chicks from 29-week-old parental flocks showed higher mortality than those from 58-week-old parental flocks. Yerpes et al. (6) reported an influence of the season on first-week mortality and emphasized the importance of controlling and minimizing seasonal fluctuations in the hatchery, on the broiler farms, or during the transport to reduce the influence of seasonal fluctuations on mortality. Although we observed differences in the mean percentages between the seasons, these variations were not significant. In the study by Yerpes et al. (6), first-week mortality was highest in fall and winter. In contrast, our findings suggested FWM to be lowest in the fall. However, the study by Yerpes et al. (6) took place in Spain, whereas our study took place in Germany. Thus, the weather conditions differ, and the differences between summer and winter may be of a higher extreme in Spain than in Germany, because of the influence of Spain's microenvironments (6). Petracci et al. (8) reported seasonal FWM percentages similar to our results, with the highest percentages in spring and the lowest in fall (8).

The average cumulative mortality in our study was 2.74% (*n* = 107 flock cycles) and was influenced by the FWM, the use of antibiotics during fattening, and the farm.

The cumulative mortality in our study was lower than the values determined in previous studies and the EFSA also states cumulative mortality of 5.00% to be usual (20). In one study, Kittelsen et al. (15) analyzed data from 59 broiler flocks of different farms and found a mean cumulative mortality percentage of 2.94%; as in our study, all broilers were Ross 308 (Aviagen, EU), and the mortality data were taken from farm evaluations. In another study, Kittelsen et al. (16) analyzed data from 61 straight run Ross 308 (Aviagen, EU) flocks, which they investigated at the processing plant, and found a mean cumulative mortality percentage of 3.0% (16). Jacobs et al. (21) and van Limbergen et al. (13) reported an overall mortality of 3.2 and 3.8%, respectively. These comparisons further support the importance of good husbandry practices to optimize the health of the flock. Our results give the impression of good management and health of the flocks evaluated in the presented study because both the average first-week mortality and the average cumulative mortality were lower than those in the studies reported.

It is reported that neonatal septicemia is one of the factors influencing overall mortality (13). In line with the findings of van Limbergen et al. (13), Tabler et al. (22), and Campe et al. (23), our analysis revealed that the cumulative mortality was influenced by the first-week mortality. Flocks with a high FWM showed higher cumulative mortality over the fattening period, which might indicate that health problems during the early stages of the life of the bird could have long-term consequences. Tabler et al. (22), for example, found that high mortality within the first days of the chicks' life resulted in a flock with poorer health status in general and with more animals susceptible to infections. Furthermore, they described a problem with uniformity in flocks with high early mortality. In the study by Campe et al. (23), mortality was additionally affected by the length of the fattening period, the hatchery, and an interaction between litter type and weather (23). In our study, the farm also influenced the cumulative mortality, thus indirectly indicating that farm-specific aspects, such as litter, hygiene, and management in general could have an influence. In line with the findings of Feddes et al. (24), neither the stocking density nor the flock size influenced the cumulative mortality in our flocks. To reduce the cumulative mortality in broiler flocks, Yassin et al. (7) recommended that farmers reduce the number of chicks, and Buragohain and Karlita (3) emphasized the importance of providing water and feed of good quality and practicing good management in the first days of the chicks' life.

The production week of the parental flock did not affect the cumulative mortality in the broiler flocks of our study, confirming the findings of Jacobs et al. (21) and Ulmer-Franco et al. (25). With regard to the season, the lowest cumulative mortality was observed in fall (2.20%) and the highest in spring (3.15%). The lowest and the highest FWM were also found in fall and spring, respectively. However, in contrast to the findings of Vieira et al. (26), no influence of seasonal differences was observed. The average slaughter weight of the Ross 308 broilers in our study was 2.30 kg (*n* = 107 flock cycles) and was influenced by stocking density, flock size, farm, season, and the interaction between antibiotic treatment and season. With an increase in the stocking density, the average weight increased as well, which is contrary to the results of Dozier et al. (27). An explanation might be, that broilers of flocks with a higher stocking density were less active due to the reduced space per bird and thus achieved a higher weight. The EU Broiler Directive 2007/43/EC requires that the maximum stocking density does not exceed 42.00 kg/m^2^ at any time (28), whereas the German Order on the Protection of Animals and Keeping of Production Animals only allows a maximum stocking density of 39.00 kg/m^2^ (5). In our study with the average stocking density of 33.86 kg/m^2^ and a range from 30.08 up to 39.43 kg/m^2^, a range of density was observed. Campe et al. (23) observed an influence of stocking density on the body weight of broilers, whereas Feddes et al. (24) found no such relationship because the mean weight of the broilers in their study did not differ between flocks of low and high stocking density. However, the flocks with the lowest stocking density in their study were less uniform than those with higher stocking densities (24). Regarding the influence of the flock size, the highest weight was found in medium flocks and the lowest weight was found in large flocks. This could be explained by the fact, that birds in larger flocks had more stress to cope with and less access to resources as already reported by Dozier et al. (27), and stress, in particular, can be the cause of reduced weight gain and reduced feed conversion (29).

The farm also influenced the average weight in our study, indicating that farm-specific differences in flock size and management could have led to those results.

There was a seasonal influence on the average weight observed in our study, with the highest weight in fall and the lowest in spring. The highest ascites findings were also observed in winter and fall, therefore, a connection could be assumed. It has already been reported that ascites can be associated with a high growth rate (30) and there was an influence of the ascites findings on the average weight in our study. Additionally, ascites can be caused by insufficient oxygenation of the body, caused by the disproportionately small heart and lungs, because the modern broilers are bred for meat yield (31) and are thus not able to cope with the higher oxygen supply over the colder months (32).

An influence of neither the FWM nor the cumulative mortality was proven in our study, which is similar to the observations of Vasdal et al. (10), who also did not find an association between the growth rate and the FWM. The authors concluded that a low FWM does not necessarily result in a faster growth rate.

The overall condemnation percentage in our study was 1.48% (*n* = 107 flock cycles), with a minimum of 0.12% and a maximum of 4.63%. Of the variables we analyzed, the season, the production week of the parental flock, and the use of antibiotics significantly influenced the condemnation percentage. The average condemnation percentage of our study is higher than those reported by van Limbergen et al. (13) (1.23%), Alfifi et al. (14) (1.10%), and Nijdam et al. (33) (0.88%). However, it is similar to the rates found by Kittelsen et al. (16) (mean 1.4%) and Santos et al. (30) (median 1.40%). The Federal Statistical Office of Germany recently stated that on average 2.10 % of the broilers slaughtered at German slaughterhouses in the year 2020 were not suitable for human consumption (33) and are thus higher than our findings.

Other studies identified the season as an influencing factor, similar to our study. Averós et al. (34) found a total percentage of carcass rejection of 0.77%, with the highest percentages in fall and spring. Salines et al. (12) found a total condemnation percentage of 1.04%, with the highest percentage in summer. They concluded that the high condemnation percentage in summer might be linked to heat, either during transport to slaughter or already on the farm (12). They also mentioned other possible influencing factors, such as the chick or feed quality (12). In our study, the highest condemnation percentages were found in fall and the lowest in winter and spring. In contrast, the mortality percentages were the lowest in fall and were high in winter and spring, and consequently, no influence was observed of either the FWM or the cumulative mortality on the condemnation percentage in our study. According to Vasdal et al. (10), a reduced condemnation percentage could be the result of poor flock uniformity, which is associated with a reduced growth rate and increased mortality rate and thus a poor general condition. An influence of the catching time on the condemnation percentage was not observed in our study. The condemnation percentage in total was also influenced by the production week of the parental flock in the same way and increased when the production week increased. This leads to the assumption that broilers from older parental flocks were more susceptible to infections which led to an increase in the condemnation percentage.

The DOA rate refers to birds that have died during their journey to the processing plant (35). In our study, the DOA percentage showed a mean of 0.17% (*n*=107 flock cycles) and was influenced by flock size, FWM, the use of antibiotics, and the interaction between antibiotic treatment and season. The mean DOA percentages reported in the literature vary greatly and range from 0.07% (15) to 0.46% (9), including reported percentages of 0.09% (16), 0.11% (36), and 0.30% (37). Flocks categorized as medium had a higher DOA percentage and the lowest DOA percentage was found in flocks that were categorized as small at the farm level. This could have been because the broilers in smaller flocks had less stress during catching and transport because especially the catching took less time. Chauvin et al. (36) and Nijdam et al. (9) also described the influence of the flock size on the DOA percentage. Bayliss and Hinton (35) described three influencing factors for the DOA percentage: health status of the flock, thermal stress during transportation, and physical injury. In our study, the FWM was shown to have an impact on DOA. With an increase in the FWM, the DOA increased as well. This could be linked to the health condition which, if it is poor, could cause high mortality during rearing and transport as the birds could not cope with the stress. Kittelsen et al. (16), on the other hand, found no differences at all in the DOA rates between flocks with low or high cumulative mortality, whereas according to Chauvin et al. (36), the DOA percentage is positively correlated with the cumulative mortality, the catching method, transportation, and weather conditions. On the contrary, Jacobs et al. (38) found a negative relationship with on-farm mortality, whereby the DOA percentage decreased by 9% with every 1% increase in on-farm mortality. We observed an influence of the catching time on the DOA rate, with higher DOA percentages found in the flocks caught in day time. This could be linked to the stress caused by heat and sunlight during the day, especially in the summer months, which the birds could not adapt to and therefore did not survive the transport. With regard to the seasons, several studies have reported an influence of the season on the DOA percentage but with different statements regarding the distribution. In the study by Averós et al. (34), the DOA percentage was highest in fall, followed by winter, spring, and summer. In the study by Petracci et al. (37), the DOA percentage was highest in summer, followed by winter, spring, and fall. Grilli et al. (39) reported a mean DOA percentage of 0.38% throughout the year, with the highest percentage in winter (0.52%) and the lowest in fall (0.22%). In our study, no influence of the season was observed.

Deep dermatitis was the main cause of condemnation (mean 0.63%, *n*=106 flock cycles) in our study and was influenced by the season and the production week of the parental flock. Dermatitis usually starts with an initial skin lesion and is followed by a secondary bacterial infection (40) with *E. coli* being the most prominent bacteria proven (41). Focal dermatitis is described as a thickening of the discolored skin, mainly unilateral, with brownish crusts. Plaques of yellowish fibrocaseous exudate could be observed on the subcutaneous tissue of the underlying skin and those lesions can mainly be found in the postventral region (41). The data evaluation of the Federal Statistical Office of Germany also name deep dermatitis as the main cause of condemnation in the year 2020 with deep dermatitis being the reason for 29.4 % of the condemned broilers (33). Similar to our results, Alfifi et al. (14) found skin disorders (scratches and dermatitis) to be the main cause of condemnation in broilers, with a prevalence of 0.24%. In addition, Salines et al. (12) found non-purulent cutaneous lesions in 20.00% of the broiler flocks and purulent lesions in 2.70%. The observed seasonal influence may have been due to the quality and moisture of the litter on the farm, a factor that is commonly associated with the season. The highest deep dermatitis findings were shown in summer, followed by fall, whereas deep dermatitis was far less frequent in winter. This could be because in the moist litter (commonly present in winter), the sharp claws of the broilers causing scratching are clogged by the litter. Tabler et al. (42) also described a higher incidence of gangrenous dermatitis in broiler flocks in summer and fall and less in winter and spring, which should be kept in check by constantly collecting the dead carcasses at the farm level. The findings of deep dermatitis in our study increased with the increasing production week of the parental flock. This could be explained by healthier birds from earlier production weeks of the parental flocks which were less vulnerable.

The other major cause of condemnation in our study was ascites (mean 0.53%, *n*=106 flock cycles). Ascites, the abdominal accumulation of fluid (43), was influenced by season, flock size, antibiotic treatment, and its interaction with season. On a national level, ascites also was found to be the second most frequent finding at German slaughterhouses and was the reason for 16.30 % of the condemned broilers in 2020 (33). Ascites is caused by an imbalance between oxygen supply and the oxygen required and results in hypoxia (44) and is the most common, non-infectious loss in the broiler industry (43, 44). Hypoxia leads to increased blood output and pulmonary hypertension, resulting in right ventricular hypertrophy, which leads to dilatation and failure of the right ventricle. The consequences are edema due to the increased blood pressure, from which the fluid leaks into the abdominal cavity (44). Ascites is known to have a genetic predisposition and can be liked to a fast growth rate (20). Other factors such as higher oxygen demand in the colder months of the year are also known to increase the risk of ascites (32). Alfifi et al. (14) recorded ascites with a prevalence of 0.22% in broiler flocks, whereas Gholami et al. (45) found cachexia as the most frequent cause of condemnation (0.15%) and ascites as a minor cause (0.03%). Salines et al. (12) found ascites in 0.10% of the flocks concerned, with generalized congestion being the most common finding (41.39% of the flocks assessed). All these reported percentages of ascites are far lower than our findings. Olkowski et al. (43) found ascites in 0.35% of the broilers condemned at slaughter and pointed out the associated risk of economic loss to the industry; the broilers with ascites are likely to die on the farm or during transport to the processing plant, and those reaching the slaughter line will be condemned. In their study, a slight seasonal trend was visible, with the incidence being highest during the colder months and lowest during summer (43). An influence of the season was also found in our study, with similar results, that is, the highest ascites percentages were found in winter, followed by the ascites findings in fall. Our results could be due to the higher oxygen demand during the colder months, as reported previously, to which the broilers' respiratory tract was not able to respond (44). Nevertheless, the ascites findings were influenced by the flock size, indicating the highest ascites findings in the flocks categorized as medium, whereas the lowest ascites findings were found in flocks categorized as small. This could be indirectly linked to the average weight, because broilers of medium flocks had the highest and small flocks the lowest weight. Therefore, a connection between ascites and the average weight can be assumed, as a relationship between ascites and growth rate has previously been reported (20, 44) and was also observed in our study within the ANOVA.

The less common causes of condemnation such as not suitable for production/general disease, hepatic changes, polyserositis, underdevelopment/emaciation, other pathologic findings/hematoma/injuries, and changes in color/odor/texture were influenced by several factors (season, cumulative mortality, stocking density, production week of the parental flock, the use of antibiotics and its interaction with season). Season was the only factor which influenced all of those dependent variables, except hepatic changes, which were only influenced by stocking density. These findings emphasize the need for maintaining good housing conditions during each season to keep the broilers in a healthy condition and thus maintain low mortality percentages. A high stocking density can be a reason for high levels of the various causes of condemnation because more birds per square meter can lead to an increase in infection pressure, stress level, and injury risk.

All broilers assessed in our study had been slaughtered at the same processing plant. Thus, the potential variance due to different assessment schemes that could lead to differences in the evaluation and documentation of slaughter results could be considered as low. Nevertheless, differences have been seen between the people who assess the birds during meat inspection (46), and also the Federal Statistical Office of Germany mentions, that although all animals are examined, some findings could not be evaluated and reported accordingly due to different recording and documentation possibilities in the slaughterhouses (33). Controlling and minimizing the use of antibiotics in Germany is of major importance, therefore, the German Federal Ministry of Food and Agriculture has implemented key points for a national antibiotic minimization concept for animal husbandry. In those, they describe that every farm with more than 10,000 broilers shall be part of the national plan to reduce antibiotic usage. Whenever an antibiotic treatment is performed, it must be documented and every 6 months those data must be sent to the HIT (central database) by the veterinarian. All data will be evaluated and whenever there is a higher usage of antibiotics than the average, special steps must be taken to improve the housing conditions and the wellbeing of the animals (47).

In our study, we furthermore considered the use of antibiotics to get an overview of the antibiotics used in the flocks observed and to investigate a possible influence on the outcomes of rearing and slaughter. One of the results we observed was that flocks with antibiotic treatment had a significantly lower cumulative mortality than flocks without antibiotic treatment. The most common causes of antibiotic treatment in our study were umbilical yolk sac inflammation (UYSI), enteritis (E), arthritis, and *E. coli* infection in broiler chicks. Antibiotic treatment was used when the mortality in a flock had increased significantly or an illness was diagnosed in the chicks. These results are in line with other publications considering antibiotic treatments of broilers (48). Other authors named neonatal septicemia to be one of the factors influencing the overall mortality (13), and the use of antibiotics would be required to control the infection. Considering the age at which the broilers were treated, 14 flocks were given antibiotics within the first week of the chicks' life, and all of these flocks were housed in spring. Treatments with Metaxol were done after the diagnosis of UYSI, whereas the other treatments done in the first week of the chick's life already started on the day the chicks were housed and after the diagnosis of enteritis. The flocks with a treatment of enteritis had a higher FWM (mean 0.69) than the flocks with antibiotic treatment because of a UYSI (mean 0.34). The Regulation (EU) 2019/6 gives information about the use of antibiotic treatments and states that antibiotic treatment must not be used routinely. It shall also not be used to compensate for poor rearing conditions and hygiene, lack of care, or poor farm management. Besides, it shall not be used to promote growth or increase yield. Prophylactic administration is only permitted in exceptional cases and only if it concerns individual animals or a limited number of animals and the risk of infection is high. In addition, the expected consequences must be severe. An antibiotic metaphylaxis is only permitted if, again, the number of animals is limited and the risk of spread of infection or infectious disease in a group of animals is high and no adequate alternatives are available (49). Two of the flocks in our study were treated with antibiotics three times and there were differences between the flocks with three antibiotic treatments with regard to the week the broilers were treated. The flock with antibiotic treatment in week 1, week 4, and week 5 had moderate first-week mortality of 0.54% (the average mean in total was 0.66%) and high cumulative mortality of 3.83% (the average mean in total was 2.74%). The flock with antibiotic treatments in week 3, 4, and 5 had first-week mortality mean value of 1.24% and cumulative mortality mean of 4.48%. Those numbers show that although health problems within the first weeks of the chick's life can be severe, diseases in the later course of the fattening period could have serious consequences.

An interaction between the antibiotic treatment and the season was visible for the FWM within the multivariate model, with differences between the season in which the treatment was done and also the number of treatments per flock. Most of the flocks that received antibiotics were treated during spring (18 of 30 flocks). Furthermore, only in spring two of the flocks were treated three times. An effect of the interaction between antibiotic treatment and season was observed on the average weight of the broilers, but for antibiotic treatment alone no effect was proven. The interaction between antibiotic treatment and season describes the seasonal differences between the flocks which had been treated and those which were not treated. Flocks without antibiotic treatment had a higher average weight, which could be linked to the better health status and a better appetite of the flocks, resulting in a better feed intake. After having discussed our results with the farm management of the two farms of our study, we were informed that the farm has noticed an increase in the flocks which needed to be treated within the first week. Therefore, it can be assumed, that our data collection was carried out during the period when the problems in the broiler fattening started and are less due to the time of the year.

The use of antibiotics during fattening also influenced the DOA percentage in our study. The mean DOA percentage in flocks without antibiotic treatment was 0.15%, whereas that in flocks that had been treated was 0.21%. The birds that had been treated were probably less resistant to stress during the transport, owing to their poorer state of health which made the previous treatment necessary.

Our analysis also showed that the records of ascites were influenced by the use of antibiotics as well as by the interaction between antibiotic treatment and season. There have been differences in the number of flocks that were treated between the seasons and this interaction was revealed within the model. Earlier infections in the broiler's life could have been a predisposing factor. *E. coli* infections, the main bacterial agent for antibiotic treatment in our study (Table 6), for example, can cause infections of the respiratory tract (55) and lead to damage. Ascites, a result of hypoxemia (44), could occur more easily as a result. In addition, Olkowski et al. (43) mention that a high percentage of broilers condemned at the slaughterhouse because of ascites can have other health issues such as cellulitis or cyanosis, which is not even reported, and cellulitis is often caused by *E. coli* infections (50), the most prominent reason for antibiotic treatments in the flocks observed. Thus, a connection between antibiotic treatment and ascites and its predisposing factors seems possible.

The interaction between antibiotic treatment and season for the DOA was also proved, which could be explained by less healthy birds, which had been treated during the rearing period. It can be assumed that the treated birds were less able to cope with the stress during catching and transport.

An influence from the interaction between antibiotic treatment and season was also found on the dependent variables ascites, polyserositis, other pathologic findings/hematoma/injuries, and changes in color/odor/texture with the highest findings being in winter in flocks that had been treated, except other pathologic findings/hematoma/injuries when the findings were lower in the flocks with antibiotic treatment. This shows that the seasonal differences between the antibiotic treatments had an effect on the slaughter results, and flocks without antibiotic treatments still were in better health at the time of slaughter.

## Conclusions

The presented multivariate analysis revealed several factors that can affect the mortality of broilers during the rearing period, their slaughter weight, and the causes of condemnation recorded at the processing plant. Cumulative mortality was influenced by FWM, antibiotic treatment, and the farm. FWM was influenced by flock size, the interaction between antibiotic treatment and season, and the production week of the parental flock. The influence of antibiotic treatment on FWM could be a result of an infection in the flock, which in many cases entails high mortality, in our study especially within the first 7 days of the chicks' life considering the differences between the seasons. Therefore, we recommend practicing special care in chick management to prevent increased losses during the fattening period and excellent hygiene to protect the health of the chicks. The average slaughter weight was influenced by the season, the stocking density, the flock size, and the farm as well as the interaction between the use of antibiotics and the seasons. The condemnation percentage was influenced by the season, the production week of the parental flock, and the use of antibiotics, whereas the DOA percentage was influenced by the FWM, the flock size, the use of antibiotics during fattening, the interaction between antibiotic treatment, and season and, in addition, by the catching time. We assume that the flocks with high FWM and the flocks that had been treated with antibiotics included animals that were less resistant to stress owing to poorer health status. Thus, they did not adapt well to the transport. The most prominent causes of condemnation (deep dermatitis and ascites) both were influenced by the season. Deep dermatitis was additionally influenced by the production week of the parental flock, whereas ascites was additionally influenced by the flock size, the use of antibiotics, the interaction between antibiotic treatment and season, and also by the average weight. Season, followed by the interaction between the use of antibiotics and season and stocking density were the independent variables, which mainly influenced the condemnation causes in our study. Although the rearing conditions are supposed to be consistent in each barn and flock throughout the whole year, there seem to be differences regarding the seasons which result in those outcomes. The stocking density could be an influencing factor because, with its increase, the infection pressure can increase similarly. The influence of antibiotic treatment alone or its interaction with the season has been observed several times in our study. This influence should be reduced by keeping excellent rearing conditions and feed and water of good quality to meet the national standards. Nevertheless, antibiotic treatment, if necessary, should be done as early as possible because infections in the flocks can increase the condemnation percentage, can lead to financial losses, and are of concern from an animal welfare perspective.

## Data availability statement

The raw data supporting the conclusions of this article will be made available by the authors, without undue reservation.

## Ethics statement

Ethical review and approval was not required for the animal study because the study exclusively included data generated after slaughter without any procedure or intervention on living animals. The data used is also generated within normal husbandry conditions in livestock.

## Author contributions

LD collected most data and transferred them to an Excel table. AJ checked the data and supplemented and completed them, conducted the statistical analysis, and prepared the first draft of the manuscript. HL commented on the previous versions of the manuscript. All authors approved the final manuscript.

## Conflict of interest

The authors declare that the research was conducted in the absence of any commercial or financial relationships that could be construed as a potential conflict of interest.

## Publisher's note

All claims expressed in this article are solely those of the authors and do not necessarily represent those of their affiliated organizations, or those of the publisher, the editors and the reviewers. Any product that may be evaluated in this article, or claim that may be made by its manufacturer, is not guaranteed or endorsed by the publisher.
